# The Autophagic Machinery in Viral Exocytosis

**DOI:** 10.3389/fmicb.2017.00269

**Published:** 2017-02-21

**Authors:** Christian Münz

**Affiliations:** Viral Immunobiology, Institute of Experimental Immunology, University of ZurichZurich, Switzerland

**Keywords:** exosome, unconventional secretion, poliovirus, coxsackievirus, epstein-barr virus, varicella zoster virus, influenza virus

## Abstract

The discovery of the molecular machinery of autophagy, namely Atg proteins, was awarded with the Nobel prize in physiology and medicine to Yoshinori Ohsumi in 2016. While this machinery was originally identified by its ability to allow cells to survive starvation via lysosomal degradation to recycle cellular components, it has recently become apparent that it also is used by cells to secrete cytoplasmic constituents. Furthermore, viruses have learned to use this Atg supported exocytosis to exit cells, acquire envelopes in the cytosol and select lipids into their surrounding membranes that might allow for increased robustness of their virions and altered infection behavior. Along these lines, picornaviruses exit infected cells in packages wrapped into autophagic membranes, herpesviruses recruit autophagic membranes into their envelopes and para- as well as orthomyxoviruses redirect autophagic membranes to the cell membrane, which increases the robustness of their envelope that they acquire at this site. These recent findings open a new exciting field on the regulation of degradation vs. release of autophagic membranes and will be discussed in this minireview.

## Introduction on autophagy

**Figure 1:**
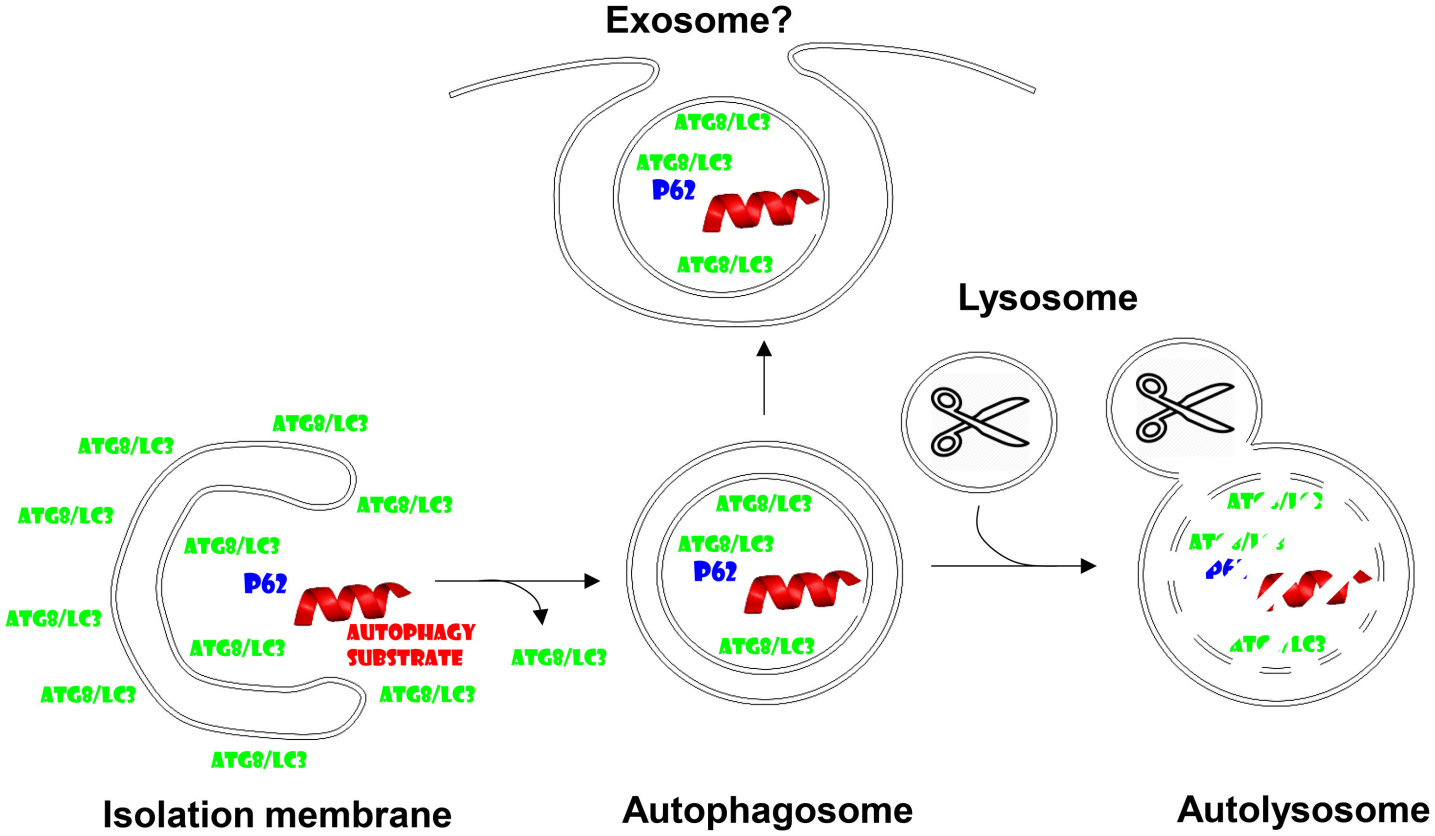
**Autophagosomes fuse with lysosomes, but might also give rise to exosome secretion**. Cytoplasmic substrates are recruited to forming autophagic membranes, the isolation membrane, via proteins that contain LC3-interacting regions (LIRs) like p62. After completion of autophagosome formation Atg8/LC3 is recycled from the outer autophagosomal membrane prior to fusion with lysosomes. Lysosomal hydrolysis degrades autophagosome cargo and the inner autophagosomal membrane. However, the inner autophagosome membrane and its content can also be secreted and might give rise to exosomes.

Autophagy or self-eating describes degradation of cytoplasmic constituents in lysosomes, which are able to break down all cellular macromolecules including lipids, polysaccharides, and proteins by virtue of their hydrolases (De Duve and Wattiaux, 1966). Autophagy summarizes several pathways, by which such macromolecules can access the lysosomal lumen from the cytosol (Mizushima et al., 2011). Macro-, micro- and chaperone-mediated autophagy are the main pathways. While micro- and chaperone-mediated autophagy perform this import directly across lysosomal or late endosomal membranes, macroautophagy generates new vesicles around its substrate. These double-membrane surrounded autophagosomes then fuse with lysosomes for degradation of the inner autophagosomal membrane and its cargo (Figure 1). However, these autophagosomes do not automatically fuse with lysosomes and I will discuss in this minireview that they can also be diverted to fuse with the cell membrane for non-canonical exocytosis, which seems to be hijacked by many viruses to acquire envelopes.

Parts of the machinery that generates autophagosomes in cells have been originally described by Yoshinori Ohsumi, the Nobel Laureate for Physiology and Medicine 2016 (Tsukada and Ohsumi, 1993). These originally named *apg* (autophagy) and later renamed *atg* (autophagy related) genes compose several functional modules in the formation of autophagosomes and their fusion with lysosomes. The Atg1/ULK1 kinase complex is under metabolic regulation, namely mTOR inhibits it under nutrient rich conditions, while AMPK activates it during starvation (Paul and Münz, 2016). The Atg1/ULK1 complex activates a type III phosphatidylinositol (PI3) kinase complex, composed of vacuolar protein sorting 34 (VPS34), VPS15, Atg6/Beclin-1, Atg14, and often AMBRA1. This complex labels membranes for autophagosome generation. The phosphatidylinositol-3-phosphate (PI3P) label recruits WIPI proteins that then serve as landing platforms for the Atg5-Atg12/Atg16L1 complex, which conjugates Atg8 to phophatidylethanolamine (PE) in the forming autophagic membrane. Prior to conjugation Atg8 is activated by C-terminal proteolytic cleavage via Atg4 and activation by the E1- and E2-like ubiquitin-like machinery of Atg7 and Atg3 proteins. In mammalian cells at least six Atg8 homologs exist, microtubule associated protein 1 light chain 3A (LC3A), LC3B, LC3C, Gamma-aminobutyric acid receptor-associated protein (GABARAP), GABARAPL1, and 2. Atg8-PE fulfills important functions in autophagic membrane elongation, which seems to be fed from Atg9 containing smaller vesicles, and substrate recruitment via LC3-interacting region (LIR) containing proteins like p62, which recruits ubiquitinated cargo to LC3 (Figure 1). Once the autophagosome closes around its cargo, presumably again via the membrane fusion activity of the Atg8 orthologues, Atg8, and Atg5-Atg12/Atg16L1 are recycled from the outer autophagosomal membrane. Autophagosomes fuse then in a Rab7 and syntaxin 17 dependent fashion with lysosomes for degradation of their cargo and the inner autophagosomal membrane (Figure 1). Nutrients like amino acids can then be recycled from these autolysosomes to sustain the growth of cells during starvation. While this mechanism has been originally described as a rather unspecific mechanism to clear cytoplasmic components during starvation, it has become clear that in most biological conditions there is a considerable hierarchy, with which organelles and protein complexes are targeted for lysosomal degradation. Along these lines starvation induces first the degradation of proteasomes, then ribosomes and only finally mitochondria (Kristensen et al., 2008), without which cell survival is not possible and complete mitophagy (autophagy of mitochondria) during extreme starvation then leads to cell death.

The above described macroautophagy pathway obviously represents a topological inversion from intra- to extracellular space, to which lysosomes belong. This inversion is similar to cotranslational transport of secreted proteins into the ER. Indeed growing evidence suggests that the macroautophagy machinery can contribute to unconventional secretion. This minireview will discuss the evidence for this alternative use of Atgs and how viruses might utilize this alternative pathway for their benefit during release from infected cells.

## Non-canonical role of autophagic proteins during unconventional protein secretion

Inefficient fusion of autophagosomes and the multivesicular bodies, to which macroautophagy contributes, with lysosomes might allow the inner autophagosomal membrane plus its cargo to be released into the extracellular space (Figure 1). This can be forced by blocking lysosomal degradation with for example lysosomal acidification inhibitors. Furthermore, proteasomal inhibition enriches defective ribosomal products (DRiPs) in such exosome like structures, which have been coined defective ribosomal products-containing autophagosome-rich blebs (DRibbles) (Yi et al., 2012). DRiPs seem to get recruited via ubiquitination and p62 mediated cross-linking to LC3 into autophagosomal membranes, which, when prevented to be degraded by lysosomes, get exocytosed (Twitty et al., 2011). DRibbles seem to be quite potent antigenic formulations for cross-presentation by antigen presenting cells (APCs) like dendritic cells. This has been documented for the cross-presentation of tumor and viral antigens (Li et al., 2011; Twitty et al., 2011; Yi et al., 2012; Ye et al., 2014; Yu et al., 2016). They can be taken up in a CLEC9A receptor-dependent manner for cross-presentation (Yi et al., 2012). As will be discussed in more detail for virus exocytosis below, the membranes of DRibbles might benefit from incorporation of autophagosome lipids and facilitate in this fashion their recognition as well as up-take by scavenger receptors on phagocytes. Along these lines cross-presentation of influenza and tumor antigens has been described to benefit from an intact autophagy machinery in antigen donor cells (Li et al., 2008; Uhl et al., 2009). This contribution of the autophagic machinery to vesicle secretion might be a more general mechanism beyond DRibbles. It has for example been described that secretory lysosomes in osteoclasts require Atgs to be released (DeSelm et al., 2011). Furthermore, exosomes released via multivesicular bodies (MVBs) seem to contain lipidated LC3 (Pallet et al., 2013). Therefore, these studies suggest that autophagosomes can be redirected to MVBs for exocytosis of their inner membrane containing cargo that has been recruited via Atg8 binding. This function obviously differs quite significantly from canonical macroautophagy and it becomes important to define if different substrates are recruited rather to this exocytosis than rather to canonical autophagy, how this is regulated and which machinery diverts these vesicles from lysosomal degradation toward secretion.

Some of these aspects have been addressed primarily with two substrates of unconventional protein secretion, namely acyl-CoA binding protein 1 (Acb1) and interleukin-1β (IL-1β). Acb1 was the first bona fide substrate for secretion that is dependent on Atg proteins (Duran et al., 2010; Manjithaya et al., 2010). This secretion of Acb1 was described to require membrane structures of Golgi origin that were termed the compartment for unconventional protein secretion (CUPS) (Bruns et al., 2011; Cruz-Garcia et al., 2014). CUPS allow the secretion of Acb1 by a mechanism that is dependent on the Golgi reassembly and stacking proteins (GRASPs) 55 and 65 as well as on MVB formation (Manjithaya et al., 2010). Also IL-1β secretion was reported to require Atgs and GRASPs (Dupont et al., 2011). Interestingly, during Acb1 and IL-1β secretion, both substrates might not be taken up into the autophagosome lumen. Acb1 might associate with CUPS membranes, which form and elongate in an Atg dependent fashion, on the cytosolic side and be transported from there to MVBs for exosome like secretion (Malhotra, 2013). IL-1β might access the intervesicular space between inner and outer autophagosomal membranes to be released after fusion of the outer membrane with the cell membrane (Zhang et al., 2015). The required translocation across the outer autophagosomal membrane seems to be dependent on HSP90 binding to KFERQ-like sequences (Q132 and Q198) in IL-1β. KFERQ-like motifs have previously been described to mediate translocation of chaperone-mediated autophagy substrates into lysosomes and late endosomes (Dice, 1990). Therefore, unconventional protein secretion seems to utilize Golgi membranes that are reshaped by the autophagic machinery. Substrates of this non-canonical pathway might associate with these membranes or even be translocated into their lumen by chaperone dependent mechanisms. In addition to Acb1 and IL-1β, also synuclein, amyloid β protein, bone morphogens, and even mitochondria have been suggested to be exocytosed in an Atg dependent fashion (Ejlerskov et al., 2013; Nilsson et al., 2013; Mankelow et al., 2015; Rosenthal et al., 2015), but it needs to be clarified if multiple exocytosis pathways use molecular components of macroautophagy or if only one pathway of autophagic exocytosis exists with many substrates. This unconventional secretion pathway seems to be hijacked by some viruses for their exocytosis, which we will discuss next.

## Autophagic envelope for non-enveloped picornaviruses

**Figure 2:**
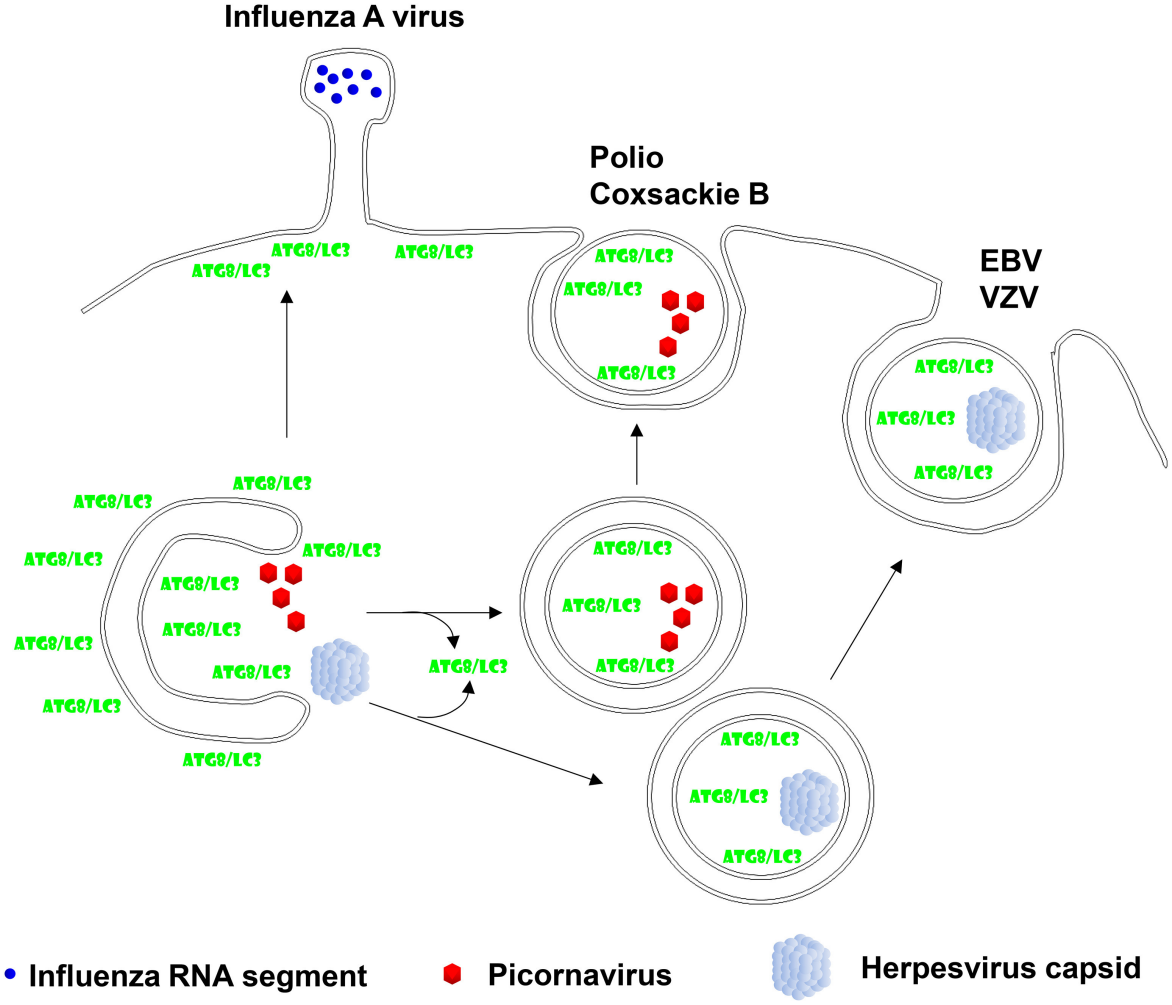
**Viruses hijack autophagic exocytosis during their release from virion producing cells**. Two of the herpesviruses, Epstein-Barr virus (EBV) and Varicella Zoster virus (VZV), have been described to acquire autophagic membranes during their second envelope acquisition in the cytosol and membrane coupled Atg8/LC3 can be found in purified virions. Non-enveloped picornaviruses, mainly polio- and coxsackie B virus have been found to exit cells in a non-lytic fashion with autophagic membranes. Finally, influenza A virus has been reported to redirect Atg8/LC3 labeled membranes to the plasma membrane in order to facilitate its filamentous budding.

The first association of autophagic membranes with a virus infection was found in poliovirus replicating cells (Dales et al., 1965). Similar to other picornaviruses poliovirus accumulates double membrane surrounded vesicles which depend on Atg8 and Atg12 for their formation (Jackson et al., 2005). The 2BC and 3A proteins of this picornavirus induce the accumulation of autophagic membranes (Jackson et al., 2005), which seem to support poliovirus release (Richards and Jackson, 2012). Indeed, non-lytic spreading of poliovirus could be inhibited by Atg8 silencing, while stimulation of autophagic membrane formation via mTOR inhibition enhanced poliovirus dissemination (Bird et al., 2014). A similar role of stabilized autophagic membranes in virus release was also found for the other picornaviruses rhinoviruses 2 and 14 as well as the foot-and-mouth disease virus (Jackson et al., 2005; O'Donnell et al., 2011). Interestingly, picornaviruses seem to even exit cells during replication in these autophagic membranes. This was first described for coxsackievirus B3 (Robinson et al., 2014). Membrane surrounded packages of these non-enveloped picornaviruses were observed to be released from infected cells. By electron microscopy these extracellular vesicles contained three to four virions. Not only lipidated LC3 was associated with these coxsackievirus containing extracellular microvesicles, but also the exosome marker flotillin-1 was found in these vesicles. This suggests that picornaviruses utilize Atg dependent exosome release as one pathway for their exocytosis (Figure 2). More recently, release in LC3 decorated membranes has now also been demonstrated for poliovirus and rhinovirus 2 (Chen et al., 2015). These poliovirus carrying vesicles contained even on average 19 virions. Their release could be inhibited by RNA silencing of Atg8 and Atg6, and stimulated by a membrane permeable tat-Atg6/Beclin-1 peptide that is thought to upregulate autophagic membrane formation by releasing Atg6/Beclin-1 from other protein associations for participation in VPS34 complexes. Therefore, picornaviruses seem to stabilize autophagic membranes and exit cells within these membranes during non-lytic spreading.

One obvious benefit for picornaviruses to surround themselves with cellular membranes is that antibodies against their adhesion receptors cannot access them during spreading. This might be especially effective, because also no viral proteins are inserted into this envelope of virus packages. More recently it has, however, been described in addition that autophagic membranes originating from ER and Golgi membranes are enriched in phosphatidylserine (PS) (Chen et al., 2015), a phospholipid that is also flipped to the outer plasma membrane leaflet during apoptosis and allows scavenger receptor mediated uptake of apoptotic bodies. Indeed, blocking of such receptors including T-cell immunoglobulin and mucin domain protein (TIM) and AXL receptor tyrosine kinase inhibits attachment, endocytosis, and infection with the respective viruses (Amara and Mercer, 2015; Chen et al., 2015). However, infection of these picornaviruses is still dependent on their specific receptors, which get exposed after presumably endosomal degradation of the surrounding autophagic membrane after up-take. Thus, non-enveloped picornaviruses surround themselves as packages with autophagosomal PS containing membranes in order to protect themselves from neutralizing antibodies and in order to utilize scavenger receptors for more efficient infection.

## Cytosolic second enveloping for herpesviruses with the help of Atgs

In addition to the above discussed picornaviruses, which are positive strand RNA viruses, also DNA viruses seem to recruit autophagic membranes to their envelope. These include the large double-stranded DNA containing coccolithovirus, which infects the oceanic alga *Emiliana huxleyi* (Schatz et al., 2014). Inhibition of PI3 kinase activity and thereby blocking of autophagic membrane generation decreased coccolithovirus production into the culture supernatant. Moreover, Atg8-II was found in virus containing fractions and could be detected by immune electron microscopy in the envelope of coccolithovirus virions.

In addition to this algal pathogen, certain herpesviruses seem to use autophagic membranes for their second envelope acquisition in the cytosol. After losing their first envelope, which herpesviruses acquire by budding through the inner nuclear membrane, via fusion with the outer nuclear membrane, they acquire ER and Golgi derived membranes for second enveloping in a process which is topologically reminiscent of macroautophagy (Johnson and Baines, 2011). The human γ-herpesvirus Epstein-Barr virus (EBV) was found to profit from autophagic membrane generation for its release during lytic replication (Granato et al., 2014; Nowag et al., 2014). EBV infection leads to the accumulation of autophagic membranes that are blocked from turnover in lysosomes. Inhibition of their generation by silencing of Atgs decreases viral particle release into the culture supernatant (Granato et al., 2014; Nowag et al., 2014). Vice versa, stimulation of autophagic membrane formation by mTOR inhibition elevates the production of infectious virions (Nowag et al., 2014). Like for the above discussed coccolithovirus, LC3B-II was found to copurify with EBV virions from the supernatant of lytically virus replicating cells (Nowag et al., 2014; Figure 2). Furthermore, LC3 could be detected by immune electron microscopy in the virus particles (Nowag et al., 2014). EBV does not only utilize macroautophagy during lytic replication, but also induces it during latent infection via its latent membrane proteins 1 and 2 (LMP1 and 2) (Lee and Sugden, 2008; Lee et al., 2009a; Fotheringham and Raab-Traub, 2015). In addition to EBV also the α-herpesvirus Varicella Zoster virus (VZV) exits cells with autophagic membranes (Buckingham et al., 2014, 2016). Pharmacological inhibition of autophagic membrane formation decreased infectious VZV production and silencing of Atg5 decreased maturation of the E glycoprotein (gE) of VZV, indicative of attenuated secondary envelope acquisition by VZV (Buckingham et al., 2014). Furthermore, gE colocalizes with LC3B and the recycling endosome marker Rab11 in VZV infected cells (Buckingham et al., 2016). Rab11 positive recycling endosomes have been reported to contribute to autophagic membranes via Atg9 and Atg16L1 dependent plasma membrane internalization (Puri et al., 2013). Interestingly, these two markers also accumulated in supernatant fractions with purified VZV virions. Particularly, lipidated LC3 co-purified with VZV virions (Figure 2). Finally immune electron micrographs cololalized LC3 with 30% of purified virions (Buckingham et al., 2016). Thus, also VZV seems to incorporate LC3 conjugated membranes into its envelope, suggesting that VZV like EBV uses the autophagic machinery to acquire its secondary envelope in the cytoplasm and incorporating the inner autophagosomal membrane into its envelope.

While the α-herpesvirus VZV and the γ-herpesvirus EBV stabilize autophagic membranes and utilize them for enveloping in the cytosol, other α-, β-, and γ-herpesviruses block macroautophagy. They inhibit Atg6/Beclin-1 recruitment into the PI3 kinase complex for autophagic membrane generation via expression of the viral proteins ICP34.5 (herpes simplex virus, HSV, α-herpesvirus), TRS1 and IRS1 (human cytomegalovirus, HCMV, β-herpesvirus) and vBcl-2 (Kaposi sarcoma associated herpesvirus, KSHV, γ-herpesvirus, and murine γ-herpesvirus 68, MHV-68; Pattingre et al., 2005; Orvedahl et al., 2007; Ku et al., 2008; Chaumorcel et al., 2012; Mouna et al., 2015). Moreover, HSV inhibits autophagy in addition to ICP34.5 with US11 (Lussignol et al., 2013), and KSHV blocks autophagy via K7 and vFLICE in addition to vBcl-2 (Lee et al., 2009b; Liang et al., 2013). Thus, many herpesviruses inhibit macroautophagy, but some (VZV and EBV) allow the formation of autophagic membrane and wrap themselves into these before leaving cells during replication. It is tempting to speculate that these later viruses profit from the particular lipids that they can recruit from autophagic membranes into their envelope, but it remains to be determined which benefits the respective lipids provide.

## Atg mediated alterations in envelope composition for RNA viruses

In addition to the above discussed direct contributions of autophagic membranes to viral envelopes and membranes for packages of non-enveloped viruses, additional RNA viruses redirect autophagic membranes to their budding sites without, however, directly incorporating Atg8/LC3 into their virions. An example for this is the influenza A virus. It blocks autophagosome maturation and fusion with lysosomes (Gannage et al., 2009). The virus achieves this inhibition of autophagosome degradation with its proton channel matrix protein 2 (M2) (Gannage et al., 2009; Beale et al., 2014; Ren et al., 2015). Recently, the proton channel activity of M2 has been directly implicated in blocking autophagosome maturation (Ren et al., 2015). The accumulating autophagic membranes seem to get in addition redirected to the plasma membrane (Beale et al., 2014; Figure 2). For this purpose M2 contains a LIR motif that is required for LC3-coated membranes to localize to the plasma membrane, the site of influenza A virus budding (Beale et al., 2014). This allows certain influenza A virus isolates to bud from filamentous membrane protrusions (Figure 2) and confers robustness against temperature mediated inactivation to the resulting influenza A virions (Beale et al., 2014). Similarly, parainfluenza virus inhibits autophagosome maturation by blocking fusion with lysosomes (Ding et al., 2014). Its phosphoprotein interacts with SNAP29 to block syntaxin 17 mediated fusion of autophagosomes and lysosomes. The accumulation of autophagic membranes in parainfluenza virus infected cells seems to support replication of this virus. Thus, influenza and parainfluenza virus seem to prevent autophagic membranes from getting degraded and redirect them to budding sites for their benefit during replication.

Another virus family that uses arrested autophagic membranes are flaviviruses (Dreux et al., 2009; Sir et al., 2012). These include hepatitis C, chikungunya and dengue virus. These viruses seem to use autophagic membranes to replicate on them in the cytosol, but also use them for their release via the exosomal pathway through MVBs (Metz et al., 2015; Shrivastava et al., 2015; Wang et al., 2015; Mohl et al., 2016). For dengue virus, pharmacological inhibition of autophagic membrane generation resulted in heat-labile virions with decreased infectivity (Mateo et al., 2013). Thus, autophagic membranes could contribute to exosomal release of flaviviruses via MVBs.

These data suggest that autophagic membranes, or more specifically their lipids, could confer robustness to both influenza and dengue virus, without LC3 however getting incorporated directly into virus particles. The nature of the respective Atg dependent lipid changes and how they affect heat-stability as well as virus infection behavior needs to be addressed in the future.

## Conclusions

Even so the autophagic machinery with its Atg proteins was originally characterized by their pro-survival role during starvation, it has recently become clear that the generated autophagic membranes do not always fuse with lysosomes for the degradation of their content. During non-canonical protein secretion and virus release from infected cells these membranes might allow cytoplasmic constituents to reach the extracellular space either in exosomes, viral envelopes or even without surrounding membranes. Some viral pathogens seem to actively block autophagosome maturation and lysosomal fusion for this purpose. These Atg supported exocytosis pathways might not only constitute one or several alternative pathways out of cells, but might also allow exosomes and viral envelopes to select distinct lipids for vesicle or virion robustness as well as for the uptake by lipid recognizing receptors. Thus, in addition to the protein machinery that forms, degrades and redirects autophagic membranes for exocytosis, the composition of these membranes should be investigated in more detail in the future. Already the observation that the inner autophagosomal membrane gets degraded by lysosomal hydrolysis, while the outer is protected from it, suggest that these two membranes that originate from one continuous isolation membrane redistribute their lipids or carefully control the lipid composition of outer and inner membrane leaflet during autophagosome formation to render the inner membrane sensitive and the outer membrane resistant to lysosomal hydrolases. It is tempting to speculate that viruses have learned to use these membrane remodeling activities to tailor their envelope.

## Author contributions

CM wrote the manuscript.

### Conflict of interest statement

The author declares that the research was conducted in the absence of any commercial or financial relationships that could be construed as a potential conflict of interest.
